# Molecular Detection of Schistosomiasis Using Real-Time PCR Before and After Treatment in Dumbi Communities, Kaduna State, Nigeria

**DOI:** 10.7759/cureus.103637

**Published:** 2026-02-15

**Authors:** Reward Muzerengwa, Iliya S Ndams, Madeline Sibula, Joshua Mbanga, Deckster T Savadye, Takafira Mduluza

**Affiliations:** 1 Research and Development, National Biotechnology Authority, Harare, ZWE; 2 African Genome Center, University Mohammed VI Polytechnic, Benguerir, MAR; 3 African Centre of Excellence for Neglected Tropical Diseases and Forensic Biotechnology, Ahmadu Bello University, Zaria, NGA; 4 Applied Biology and Biochemistry, National University of Science and Technology, Bulawayo, ZWE; 5 Centre for Immunology and Infection Research, Biotechnology and Biochemistry Department, University of Zimbabwe, Harare, ZWE

**Keywords:** conventional pcr, microscopy, praziquantel (pzq), real-time pcr, schistosoma haematobium

## Abstract

Background

Schistosomiasis, a neglected tropical disease, is a major helminth disease in terms of morbidity and mortality. This study assessed the use of real-time PCR for the detection of *Schistosoma* *sp*. DNA in both urine and faeces samples before and after praziquantel (PZQ) treatment, and the results were compared with those of conventional PCR and microscopic detection of schistosome eggs to evaluate treatment efficacy.

Materials and methods

A community-based cross-sectional study was carried out from August 2020 to January 2021 in Dumbi Communities of Nigeria. Both urine and stool samples were collected from three hundred and eighty-seven (387) study participants aged between three and 25 years before and after (three weeks and eight weeks) treatment with PZQ. On the treatment day, all participants who tested positive after microscopic examination in the community were treated with a single dose of 40 mg/kg PZQ. DNA was isolated from 50 samples (urine), including those that tested positive using microscopy as part of initial screening.

Results

Utilising diagnostic methods, microscopy detected *Schistosoma haematobium* eggs in 7.5% (n=29) of the urine samples collected before treatment, whereas real-time PCR amplified DNA in 39.8% (n=154) of the same samples, and no eggs were detected in the stool samples analysed. Among the diagnostic methods for 50 urine samples that were used for comparative analysis, real-time PCR had the highest positive detection of 80%, followed by conventional PCR (72%), haematuria (64%), and microscopy (58%). Compared with microscopy, real-time PCR and conventional PCR both provided lower estimates of cure rates.

Conclusions

The results of this study revealed that real-time PCR and conventional PCR are significantly more sensitive than microscopy for detecting and evaluating infection incidence, which is an important aspect of epidemiological studies. The real-time PCR-based detection technique can be especially useful in circumstances of lower intensity or prevalence of infection, a condition for which the parasitological diagnosis shows a well-proven limitation of its sensitivity.

## Introduction

Schistosomiasis is a neglected tropical disease (NTD) caused by parasitic trematodes of the genus *Schistosoma* and is prevalent in sub-Saharan Africa (SSA), Asia, and Latin America [1]. Schistosomiasis, a major human helminthic disease in terms of morbidity and mortality, affects more than 230 million people globally [2]. The groups of people most affected in terms of morbidity are young adults and school-aged children (SAC) [3]. In SSA, the two major types of schistosomiasis are caused by *Schistosoma haematobium* (urogenital schistosomiasis) and *Schistosoma mansoni *(intestinal schistosomiasis) [4]. Regular chemotherapy with praziquantel (PZQ) is required to control severe morbidity [5].

According to Utzinger et al. [5], if the World Health Organisation (WHO) is to treat at least 75% of all infections in school-aged children, then an estimated 128 million school-aged children need PZQ treatment each year for rudimentary control of schistosomiasis. Between 2010 and 2019, the total number of people treated increased substantially from 33 to 105.4 million, and the number of tablets from the 2020 campaigns was estimated at 226 million [1]. Although globally efficient, this ongoing and repeated mass drug administration (MDA) acts as an important drug pressure, which could lead to the emergence of schistosome strains that are resistant to the PZQ [2,6]. In order to properly identify those infected and assess chemotherapy in field settings, schistosomiasis must be controlled using diagnostic techniques that are dependable, precise, accurate, specific, and sensitive [7,8].

The majority of epidemiological evaluations of the burden of schistosomiasis have depended on microscopic detection of both intestinal and urinary schistosomes [9]. A comparatively simple and affordable tool for identifying and calculating the concentration of schistosome eggs in faecal and urine specimens is offered by this technique in many developing nations where schistosomiasis is endemic [10]. However, due to false-negative scenarios, the challenges of fulfilling the multiple sampling requirements for traditional parasitological diagnosis have frequently resulted in less-than-ideal outcomes [5]. Antibody-based assays are sensitive, but they are unable to distinguish an active infection from the history of exposure. Additionally, they have been shown to exhibit cross-reaction with other helminths, and they are not readily implementable under field settings [11]. These factors have limited the applicability of antibody-based detection assays in SSA, particularly in regions where the disease is highly endemic and residents are at risk of re-infection after treatment [4,12]. Antigen-based assays, such as circulating cathodic antigen detection in urine, have proven to be useful, field-applicable techniques for detecting *S. mansoni* infections, though the test has been shown to be less sensitive for infections with *S. haematobium* [13].

Polymerase chain reaction (PCR) methods have demonstrated high sensitivity and specificity for the identification of parasitic DNA and have been successfully employed to reveal the presence of DNA from a wide range of parasites, including *Schistosoma* sp. [14,15]. Its high sensitivity enables the diagnosis of schistosomiasis even when no eggs are found by microscopy. This situation can occur during the early stages of infection, when worms have not yet produced large numbers of eggs [16]. It is also relevant during active and chronic infections with low egg counts [16,17], as well as in cases where egg excretion is low, as seen in immunocompromised individuals [9,18]. A vital advancement made by real-time PCR is the relatively timely production of results for many samples and the early detection of amplicons via sensitive fluorescence detection equipment [19].

PCR can be employed to assess MDA programs by detecting residual infections that microscopy might miss [20]. PCR methods are also of paramount importance in identifying asymptomatic carriers who can harbour infection, which is important for breaking transmission cycles [21]. In scenarios where selective treatment is needed, for example, in high-risk populations, PCR can identify individuals who require treatment, optimising the use of resources [3]. When it comes to cost-effectiveness factors, while MDA decisions are made at the population level, the sensitivity of the PCR assays can influence the overall effectiveness of control programs [2]. The *Schistosoma *species that is prevalent in the Dumbi community, Kaduna State, Nigeria, is *S. haematobium,* and the real-time PCR that was applied was species-specific. Therefore, the goal of this project was to employ a real-time PCR-based detection method for schistosomiasis before and after treatment in SAC and young adults (3-25 years old) in the Nigerian study area.

This article was previously posted to the Research Square preprint server on November 20, 2024.

## Materials and methods

Study design

A community-based cross-sectional study was carried out from August 2020 to January 2021. Before the recruitment of children into the study, the objectives of the study were explained to both the parents and community leaders, and the participation required in the study was indicated. A random sampling procedure was used to recruit the 387 participants who volunteered to provide their samples. The study consisted of parasitological baseline screening, treatment of children with a single oral dose of PZQ (40 mg/kg), follow-up surveys at three and eight weeks of treated participants, especially those infected at baseline, DNA extraction, and PCR amplifications of *Schistosoma *spp. DNA from samples collected at each time point was used to compare methods and evaluate treatment efficacy.

Study area and sample collection

**Figure 1:**
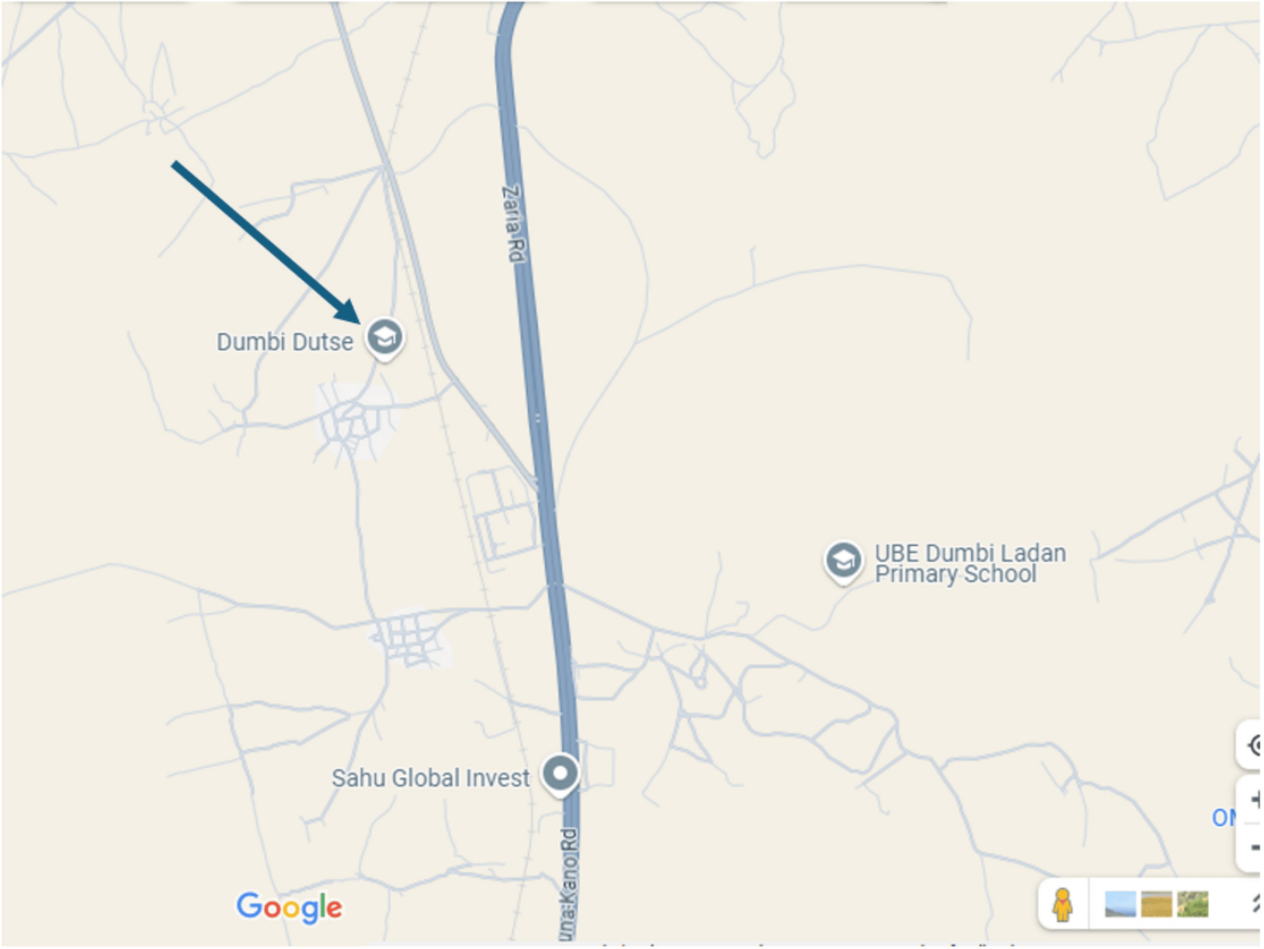
Map of Igabi local government area (LGA) of Kaduna state, Northern Nigeria, showing the Dumbi study community Created from Google Maps (Google, Inc., Mountain View, US) and edited using Microsoft Word (Microsoft Corp., Redmond, US).

The study was carried out in Dumbi, one of the clusters of villages situated in the Igabi Local Government Area of Kaduna state, northern Nigeria, which is located between longitude 70 37'E and latitude 100 56'N. Dumbi comprises six settlements, three of which were used for this study: Dumbi Ladan, Dumbi Kastinawa, and Dumbi Dutse (Figure 1). Urine and stool samples were collected in 50 ml containers from all (387 urine and 50 stool samples) registered participants between 10:00 and 14:00, when schistosome egg excretion is known to be highest, and were left at ambient temperature between the collection sites and the Zoology Department laboratory of Ahmadu Bello University (ABU). This process complied with institutional approvals obtained from the African Centre of Excellence in Neglected Tropical Diseases and Forensic Biotechnology Research Board and the Ahmadu Bello University Committee of Human Subjects for Research (Reference Number: ABUCUHSR/2023/044). Each urine sample was tested for haematuria, proteinuria, glucose, and pH using Swe-Care Urine Reagent Strips (Nantong Diagnos Biotechnology Co. Ltd, Nigeria) for urinalysis (URS-9). The presence and quantification of *S. haematobium* ova were measured using the sedimentation method. Briefly, the tubes containing urine were shaken to resuspend the eggs, and 10 ml of each collected urine sample was concentrated through centrifugation. Each of these urine samples was spun for five minutes at 4500 rpm in a centrifuge. After which, the supernatant was discarded, and the sediment was examined at 10x magnification under a light microscope. Eggs were easily detected and identified by shape and the position of the spine, which was terminal for *Schistosoma haematobium* [22]. Intensity of infection was recorded as egg number per 10 ml of urine. The slides were analysed microscopically for schistosome eggs. For each subject, the urine sample was examined for ova of *S. haematobium*, which allowed the intensity of infection to be expressed as the number of *S. haematobium* eggs/10 mL urine, with counts of 1-50 and ≥50 eggs/10 mL indicative of low and high intensity of infections, respectively. A 2 ml aliquot of each urine sample was kept frozen at -80°C for PCR analysis.

Stool specimens collected from each individual were prepared for microscopy examination using the saline gradient method. Briefly, 0.5 g of stool was taken with a wooden spatula and scraped through a plastic mesh sieve of pore size 105 μm to remove particulate and fibrous material. A hole in a plastic template set on a glass microscope slide was filled with the specimen. The template, when filled, contained approximately 41.5 mg of faeces. The template was lifted carefully off the slide, and the stool specimen cast on the slide was covered with a 25x35 mm cellophane coverslip impregnated with saline water. The slide was turned upside down on a flat surface and pressed gently, but firmly, to spread the stool specimen evenly under the cellophane. The slide was left at room temperature for 30 minutes to clear before it was examined under the microscope for eggs of *Schistosoma *sp. and any soil-transmitted helminths present. Two slides were prepared for every sample and examined by two different technicians. The average egg count from the two slides was recorded as egg count per that sample, and the egg count over the two days for each participant was also averaged and expressed as egg per gram (EPG=egg countx24). With this, egg counts were categorised into egg intensity classes: negative, light intensity (1-100 EPG), moderate intensity (101-400 EPG), and heavy intensity (>400 EPG) [15].

Sample size determination

The sample size was determined using the Fisher formula: n=Z^2^pq/D^2^, ​where n is the minimum sample size, Z is the standard normal distribution at 95% confidence interval (1.96%), p shows the prevalence rate, which is taken as 25% (0.25) [23], q=1-p=0.75, and allowable error D is set as 95%=0.05. 

Based on the formula above, a minimum of 330 samples were estimated, and 387 samples were collected from consented participants.

Inclusion and exclusion criteria

Study participants aged from three to 25 years (school-aged children and young adults) living in Dumbi communities for at least one year were included in the study. Individuals who had taken praziquantel treatment six months before the study and who had other chronic conditions, such as chronic kidney disease and diabetes mellitus, were excluded from the study.

Data collection

During the baseline study, interviewer-administered questionnaires were used to collect sociodemographic information from participants. A simple questionnaire was used to collect information such as sex, age, type of sample, and village of residency (see Appendix 1). A short interview was conducted among participants who provided stool and urine samples only. Participants were fully informed about the nature of the study and provided their consent prior to enrolment. 

Treatment of participants and post-treatment sampling

On the treatment day, all participants who tested positive after microscopic examination in the community were treated with a single dose of 40 mg/kg PZQ (PZQ USP 600 mg; Chez Resources Pharmaceuticals Limited, Nigeria), as recommended by the WHO treatment strategy for schistosomiasis [1]. Only 29 (7.5%) of the children sampled at baseline were part of the treatment group. After drug administration, three-week and eight-week follow-up parasitological sampling was performed. Urine and stool samples were collected from 29 participants who were sampled at baseline and treated at each follow-up time point. In the laboratory, 10 ml aliquots of urine were processed for microscopy analysis after urine analysis (glucose, protein, pH, and blood levels) had been done, and 2 ml were kept frozen at -20 °C for molecular analysis. Approximately 0.5 g of each stool sample was analysed via the saline gradient method, and an aliquot of approximately 1 g of each sample from each person was kept frozen in cryotubes at -20°C for molecular analysis.

DNA extraction

DNA was isolated from urine samples, including those that tested positive using microscopy as part of initial screening (the additional urine samples extracted were negative on microscopy and were included through random sampling). No DNA extraction from stool samples was done since the 50 stool samples analysed had tested negative at microscopy as part of initial screening. DNA extraction was performed using the Zymo DNA Mini Extraction Kit (Inqaba Biotec, Western Africa) and the QIAamp® DNA Mini Kit (QIAGEN, Hilden, Germany) following the manufacturers' protocols (Appendix 2). Before extraction, a 200 µl subsample of each centrifuged urine sample was heated for 10 min at 100°C before being treated with sodium dodecyl sulfate and proteinase K for 2 h at 55°C [24]. Phocin herpes virus 1 (PhHV-1), which was used as an internal control, was added to the lysis buffer (1000 plaque-forming units/ml), which was obtained from the Biochemistry Department of ABU, Zaria, Nigeria.

Conventional PCR and gel electrophoresis

The primers used were obtained from Inqaba Biotechnical Industries, Western Africa. PCR was carried out using a forward primer Ssp48F (5'-GGTCTA GAT GAC TTG ATY GAG ATG CT-3'), reverse primer Ssp124R (5'-TCC CGA GCGYGT ATA ATG TCA TTA-3'), and *Schistosoma* genus-specific primers, which amplified a 77-bp fragment of the internal transcribed spacer-2 (ITS2) subunit [14]. PCR was also carried out using a forward primer Sh307F (5'-CCT CCA TTA TCC ATA TCT GAG AAT TCA-3') and reverse primer Sh447R (5'-AGA AGT CTT AAA ATC GAC ACG ACT AAT AAT C-3') to amplify a 143-bp region with a highly repetitive sequence in *S. haematobium* targeting the 18S ribosomal DNA (rDNA) intergenic spacer region. For amplification, 5 μl of the DNA served as the PCR template. The total reaction volume was 25 μl, consisting of 0.5 µl of Taq DNA polymerase (1.25 units), 2.5 µl of 10x buffer (1.5 mM), 1.5 µl of 1.5 mM MgCl2, 0.5 µl of dNTPs, 0.5 µl of each of the amplification primers (50 pmol), and 14 μl of nuclease-free water to complete the final volume. The amplification profile was performed in steps of initial denaturation at 95°C for 15 minutes followed by 30 cycles of denaturation at 95°C for 30 seconds, annealing at 58°C for one minute, and elongation at 72°C for 30 seconds, followed by a final extension step at 72°C for five minutes in a Gene Amp Thermal Cycler System 9700 (Applied Biosystems Co., Waltham, US). From the PCR run, 5 µl of each of the 50 samples (50 DNA samples from urine) were mixed with 4 µl of the 6x loading dye (0.255 bromophenol blue, 0.25% xylene ethanol, 30% glycerol) and loaded into each well of the gel. A 1% (w/v) high-resolution agarose gel stained with 5 µl of ethidium bromide was used for analysis of the products. After electrophoresis, the gels were visualised with an imaging system (documentation system from UVIteck Ltd., Cambridge, UK), and the digital images were captured.

Real-time PCR

*Schistosoma* genus-specific primers amplifying a 77-bp fragment of the internal transcribed spacer-2 (ITS2) sub-unit described by Obeng et al. [14], consisting of Ssp48F (5'-GGT CTA GAT GAC TTG ATY GAG ATG CT-3') and Ssp124R (5'-TCC CGA GCG YGT ATA ATG TCA TTA-3'), detected by the probe, Ssp78T (FAM-5'-TGG GTT GTG CTC GAG TCG TGGC-3'-Black Hole Quencher; Biolegio), were chosen for amplification of the presence of *Schistosoma* DNA. The amplification of each DNA sample was performed in a 25 µl reaction mixture containing PCR buffer (HotstarTaq mastermix; QIAGEN), 5 mM MgCl2, 12.5 pmol of each *Schistosoma *genus-specific primer, 15 pmol of each PhHV-1-specific primer, 2.5 pmol each of the *Schistosoma* genus-specific and 1 µl of PhHV-1-specific double-labelled probes, and 5 µl of the DNA sample. The qPCR machine employed was set to give three minutes at 95°C, followed by 50 cycles of 15 seconds at 95°C, one minute at 58°C, 30 seconds at 72°C, and 5 seconds at 72°C. Amplification, amplicon detection, and related data analysis were performed with a CFX96 real-time PCR detection system (Bio-Rad, Veenendaal, Netherlands). CFX Manager Version 1.6.514 (Bio-Rad) was used for related data analysis. The PCR output from this system consisted of a cyclic threshold (Ct) value, representing the amplification cycle in which the level of the fluorescence signal exceeds the background fluorescence, indicating the parasite-specific DNA load in the urine sample tested. A test was considered positive when the Ct value was less than 50 and greater than 15, considering the nature of the amplification curve. For identification of *S. haematobium*-specific DNA, the primers Sh307F and Sh447R were chosen to amplify a fragment of 143 bp.

Data analysis

Statistical analysis was done using the SPSS software package (IBM SPSS Statistics for Windows, version 20.0, Armonk, US) after tabulation of epidemiological data in MS Excel 2016 (Microsoft Corp., Redmond, US). Real-time PCR results were stratified into high (Ct<30), moderate (30≤Ct≥35), low (Ct>35) DNA load, and negative (no amplification detected in 50 cycles). The positive and negative predictive values of microscopy and real-time PCR were calculated with true positives as microscopy and/or PCR positive and microscopy and PCR negatives assumed to be true negatives. A participant was defined as positive if *Schistosoma* spp. eggs were found in urine and/or stool, and was defined as negative when there were no eggs in both samples. Data analysis was performed on populations of size 387. All tests were performed at a significance level of 0.05.

## Results

Microscopic examination before treatment

**Figure 2:**
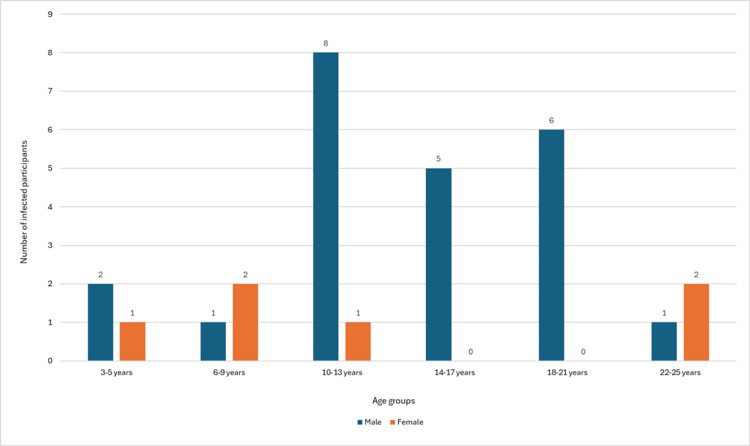
Schistosoma-infected participants at the baseline among age groups and sexes (n=29)

Among the 387 participants registered and sampled, 29 (7.5%) had *Schistosoma* eggs (*S. haematobium*) present in their urine samples (Table 1). There were no *Schistosoma* eggs in the 50 stool samples that were collected. Infection at the baseline among different age groups and sexes is represented in Figure 2.

**Table 1:** Urine microscopy results of the participants before treatment

Microscopy analysis	Male, n (%)	Female, n (%)	Total, n (%)
Number sampled	181 (47)	206 (53)	387 (100)
Number infected	23 (5.9)	6 (1.6)	29 (7.5)
Intensity of infection			
Light infection: 1-49 egg/10 ml	20 (70)	6 (20.7)	26 (89.7)
Heavy infection: ≥50 eggs/10 ml	3 (10.3)	0	3 (10.3)
Types of schistosome egg found			
*S. haematobium* only	23 (79.3)	6 (20.7)	29 (100)

Urogenital schistosomiasis prevalence and intensity three weeks after treatment

Among the 25 treated participants, five males were egg positive after three weeks of re-examination. Overall, an 80% cure rate was observed (Table 2). The other four participants who were egg positive did not show up at the treatment stage.

**Table 2:** Prevalence and intensity of eggs in urine three weeks after treatment

Egg intensity	Number re-examined			Number infected			Cure rate		
	Male	Female	Total	Male	Female	Total (%)	Male	Female	Total (%)
1-49 eggs	16	6	22	5	0	5 (22.7)	11	6	17 (77.3)
≥50 eggs	3	0	3	0	0	0 (0)	3	0	3 (100)
Total	19	6	25	5	0	5 (20)	14	6	20 (80)

Infection status eight weeks after treatment

Upon the treatment of the five infected patients after the first three weeks, now into week 8, there was a noted 100% cure rate. The earlier 5 infected patients had fully recovered (Table 3).

**Table 3:** Infections in urine eight weeks after treatment

Age groups	Number of treated and re-examined			Number of infected			Cure rates
	Male	Female	Total	Male	Female	Total	Total (%)
3-5	1	1	2	0	0	0	2 (100)
6-9	2	1	3	0	0	0	3 (100)
10-13	5	1	6	0	0	0	6 (100)
14-17	3	0	3	0	0	0	3 (100)
18-21	4	0	4	0	0	0	4 (100)
22-25	0	2	2	0	0	0	2 (100)
Total	15	5	20	0	0	0	20 (100)

Conventional PCR amplification prevalence before treatment

**Figure 3:**
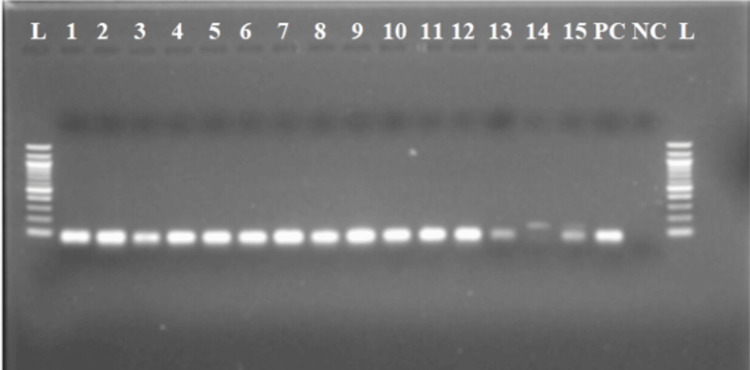
Conventional PCR amplicons from urine samples before treatment (Lane 1-15) NC - negative control (ultra-pure water), PC - positive control (Schistosoma DNA-Qiagen), L - 1000 bp ladder

From the 50 samples whose baseline was tested employing conventional PCR (Figure 3), a total of 36 individuals were found to be positive. There was no significant relationship between the age group and sex parameters of the 36 participants who tested positive according to the conventional PCR test (χ^2^=6.222, p=0.285).

Cure rates as measured by conventional PCR

Taking note of the 25 treated egg-positive participants who were re-examined after treatment, conventional PCR found *Schistosoma *infection in five (20%) of the urine specimens. This resulted in an 80% cure rate. The PCR tests at eight weeks found 0% (out of 25) of the infection in the specimens. That resulted in a 100% cure rate (Table 4).

**Table 4:** Cure rates as measured by conventional PCR

Age groups	Number of treated and re-examined			Number of negatives at three weeks after treatment			Number of negatives at eight weeks after treatment		
	Male	Female	Total	Male	Female	Total (%)	Male	Female	Total (%)
3-5	1	1	2	1	1	2 (100)	1	1	2 (100)
6-9	2	1	3	2	1	3 (100)	2	1	3 (100)
10-13	6	1	7	5	1	6 (85.7)	6	1	7 (100)
14-17	5	0	5	3	0	3 (60)	5	0	5 (100)
18-21	6	0	6	4	0	4 (66.7)	6	0	6 (100)
22-25	1	1	2	0	2	2 (100)	1	1	2 (100)
Total	21	4	25	15	5	20 (80)	21	4	25 (100)

Cure rates as measured by real-time PCR

Taking note of the 25 treated egg-positive participants who were re-examined after treatment, just like conventional PCR, real-time PCR found *Schistosoma *infection in five (20%) of the urine specimens. This resulted in an 80% cure rate. The PCR tests at eight weeks found 0% (out of 25) of the infection in the specimens. That resulted in a 100% cure rate (Table 5).

**Table 5:** Cure rates as measured by real-time PCR

Egg intensity	Number of treated and re-examined			Number of negatives at three weeks after treatment			Number of negatives at eight weeks after treatment		
	Male	Female	Total	Male	Female	Total (%)	Male	Female	Total (%)
1-49 eggs	18	4	22	13	4	17 (77.3)	18	4	22 (100)
Over 50 eggs	3	0	3	3	0	3 (100)	3	0	3 (100)
Total	21	4	25	16	4	20 (80)	21	4	25 (100)

Comparison of microscopy and real-time PCR detection of Schistosoma infection

From all the 387 participants whose urine samples were collected and analysed in 2021, specimens that revealed higher egg intensity employing microscopy were better detected by real-time PCR compared to those that revealed lower egg intensity (Table 6). As low as three (100%) of the high egg counts (>50 eggs per 10 ml urine) showed low Ct values (Ct<30), indicating a higher amount of schistosome DNA in the specimens. No specimens had relatively high Ct values (above 35 cycles). None of the high-intensity samples tested negative for real-time PCR, indicating 100% sensitivity of high infections (>50 eggs per 10 ml urine) to real-time PCR detection. Out of the 358 negative individual samples captured by the microscopy egg count, 125 of them (34.9%) tested positive for schistosome DNA. Out of the 26 individuals that were detected to be of low-intensity infections (<50 eggs per 10 ml urine) microscopically, no one tested negative for real-time PCR.

**Table 6:** Comparison of microscopy and real-time PCR detection of Schistosoma infection Ct - cyclic threshold

Microscopic egg count	Real-time PCR class				
	Negative	Ct>35	30 < Ct < 35	Ct<30	Total
Negative	233	45	45	35	358
1-49 eggs/10 ml	0	4	8	14	26
≥50 eggs/10 ml	0	0	0	3	3
Total	233	49	53	52	387

Comparative evaluation via microscopy, haematuria, conventional PCR, and real-time PCR

Compared with the diagnostic methods for 50 urine samples that were used for comparative analysis (Table 7), real-time PCR had the highest percentage of positive samples (80%), followed by conventional PCR (72%), haematuria (64%), and microscopy (58%). 

**Table 7:** Comparative evaluation of the microscopy, haematuria, conventional PCR and real-time PCR testing

Diagnostic test	Number of positives	Number of negatives	Total	% of positives
Microscopy	29	21	50	58
Haematuria	32	18	50	64
Conventional PCR	36	13	50	72
Real-time PCR	40	10	50	80

## Discussion

The results from this study revealed a significant difference in the prevalence of infection (*S. haematobium* and/or *S. mansoni*) in the study area (p=0.05) between microscopy (7.5%) and real-time PCR (39.8%) among the 387 participants whose urine samples were collected before treatment. This is closely comparable to a report from South-West Nigeria, which gave 19% and 9% for urogenital and intestinal schistosomiasis, respectively [20]. Among the 50 stool samples tested via microscopy, no eggs were found in the participants tested, which shows that *S. mansoni* is not common in the Dumbi Community, Nigeria [23]. There are no published reports of *S. mansoni* infection in Dumbi. However, the absence of the helminth in the community aligns with the study reports in Odeda LGA that examined the spatial distribution of schistosomiasis in Ogun State [25]. We did not conduct snail sampling, but our findings may be partially because of the absence of the snail intermediate host, *Biomphalaria peifferi*, in the Kaduna River, the primary source of water for the community and the site of schistosomiasis. The results of real-time PCR and conventional PCR were also similar, as conventional PCR had a positive detection rate of 72%, which identified 36 positive cases from the 50 urine samples analysed by the *Schistosoma* genus-specific primers. The drastic reductions in infection intensity and prevalence following treatment with PZQ, as shown by the 80% cure rate and 100% cure rate at three and eight weeks post-treatment, respectively, by microscopic detection of eggs in urine and/or stool samples, with no significant differences between sexes and age groups, are supported by a previous study done in Senegal by Senghor et al. [2], who reported similar reduction rates in prevalence after three to four weeks post-treatment with PZQ. Real-time PCR and conventional PCR had similar cure rates, with rates of 80% and 100% detected at three weeks and eight weeks post-treatment using* Schistosoma *genus-specific primers, as noted with microscopy.

This prevalence of infection at baseline by both diagnostic methods was characterised by a classical increase in both the prevalence and intensity of infection with increasing age, which peaked in children aged between 10 and 13 years, similar to reports in Cote d'Ivoire [26], Ghana [21,27], and Nigeria [28]. The contribution of age to the risk of schistosomiasis may not act independently. Instead, it might be interconnected with other factors influencing childcare, such as parental and social status and educational level [29]. This is due to the fact that children engaging in activities, such as swimming and playing in contaminated water sources, could be dependent on parental supervision, which, in itself, is affected by economic status.

The higher sensitivity of the real-time PCR method and conventional PCR at all three time points investigated is supported by the ability of the PCR technique to amplify a minute amount of DNA exponentially to a larger volume that can be detected and even quantified, and is consistent with previously reported underestimation of the prevalence of infection by microscopic detection of eggs compared with PCR detection [21].

All 387 participants' urine samples were collected and analysed, and specimens that revealed higher egg intensity employing microscopy were better noted by real-time PCR compared to light intensity. As many as three of the greater than 50 eggs per 10 ml of urine (high egg count) had a low Ct value (Ct<30), suggesting that more schistosome DNA was present in the sample. None of the samples had considerably more than 35 cycles (high Ct values). Additionally, none of the more than 50 eggs per 10 ml of urine (high egg count) samples yielded negative real-time PCR results, suggesting high sensitivity of infection (more than 50 eggs per 10 ml of urine) to real-time PCR detection. Among the 358 negative specimens detected by microscopic egg counting, 125 (34.9%) were positive for schistosome DNA. Of the 26 individuals who were microscopically found to have fewer than 50 eggs per 10 ml of urine (low-intensity category), none tested negative by real-time PCR. The results of this study support a number of other reports [14,30], which also indicated 100% detection of high-intensity egg counts by real-time PCR amplification. However, four urine samples with light egg intensity (diagnosed by microscopic egg count) were missed; these samples were picked by real-time PCR and considered negative by conventional PCR, which can be explained by the presence of insufficient schistosome DNA in the isolated samples for positive detection [14] and the possibility of variation in egg output and uneven distribution in the samples [2]. These results demonstrate that diagnostic sensitivity can be increased using molecular techniques.

In our study, there were some limitations on the sample size as we had to streamline and reduce the number of samples down to 50 for the molecular analysis, where comparative analysis was done with other methods due to limited resources. Another issue to note was that only 50 stool samples were provided from the 387 participants, which could have been due to socioeconomic factors and general acceptability when it comes to faecal collection, and we could not proceed to do molecular analysis of the stool samples since, at initial screening, there was no positive egg detection. An assumption was also made that *S. mansoni* was not endemic in the Dumbi community [23]. After microscopy, analysis for four participants from the 29 participants that had tested positive for microscopy did not show up at treatment day and thus, were not treated, so we had to work with 25 participants for post-treatment analysis. The comparison of the diagnostic methods did not include an analysis of concordance or discordance among the methods. Instead, it only reported the number of positive and negative cases, as well as the totals and percentages. This analysis could have been a valuable addition to the study.

## Conclusions

The results of this study reveal that real-time PCR can serve as a powerful alternative for determining the prevalence and intensity of *Schistosoma* infections using urine samples as templates. This can lead to a more accurate assessment of the prevalence and intensity of the disease and the evaluation of the effectiveness of treatment programs in endemic areas. Molecular characterisation of the identified *Schistosoma *spp. is recommended, especially for treatment monitoring in cases of reinfection and *Schistosoma-*resistant strains, which may be identified. Next-generation sequencing (NGS) of the *Schistosoma *spp. is also recommended for molecular phylogenetic analysis. There is a need to conduct ongoing assessments in the Dumbi community, using larger cohorts and data from surrounding regions in Kaduna State. There is also a need for the development of biomarkers for bladder cancer detection in females infected with urogenital schistosomiasis, which can be complemented by sensitive and specific PCR-based methods.

## Appendices

Appendix 1. Dumbi community Schistosomiasis questionnaire 2020

Name  

Sex  

Village  

Unique ID Number  

Source of water  

Occupation/Class  

Appendix 2. Quick DNA Miniprep Plus K: biological fluids and cells extraction protocol

Add 1060 µl of storage buffer to each 20 mg tube of proteinase K. Store at -20˚C.

1. Add up to 200 µl sample to a microcentrifuge tube and add 200 µl BioFluid & Cell Buffer (red) and 20µl proteinase K. Note: for inputs <200 µl biological fluid, proportionally decrease BioFluid & Cell Buffer (red), proteinase K and Genomic Binding Buffer.

2. Mix thoroughly and then incubate the tube at 55˚C for 10 minutes.

3. Add 1 volume Genomic Binding Buffer to the digested sample. Mix thoroughly. Example: add 420 µl Genomic Binding Buffer to the 420 µl digested sample.

4. Transfer the mixture to a Zymo-SpinTM IIC-XL column in a collection tube. Centrifuge (≥12000xg) for one minute. Discard the collection tube with the flow-through.

5. Add 500 µl DNA pre-wash buffer to the column in a new collection tube and centrifuge for one minute. Empty the collection tube.

6. Add 700 µl g-DNA wash buffer and centrifuge for one minute. Empty the collection tube.

7. Add 200 µl g-DNA wash buffer and centrifuge for one minute. Discard the collection tube with the flow-through.

8. To elute the DNA, transfer to a clean microcentrifuge tube. Add ≥50 µl DNA elution buffer, incubate for five minutes, and then centrifuge for one minute.
